# Posterior cruciate ligament reconstruction with independent internal brace reinforcement: surgical technique and clinical outcomes with a minimum two year follow-up

**DOI:** 10.1007/s00264-022-05448-4

**Published:** 2022-05-26

**Authors:** Xin Zhao, Ming Yi Duan, Si Qi Chen, Junyuan Wang, Wenxuan Li, Yuhang Lv, Hang Zhou Zhang

**Affiliations:** 1 Department of Operating Theatre, Joint Surgery and Sports Medicine, First Affiliated Hospital of China Medical University, 155 Nanjing North StreetLiaoning Province, Shenyang, 110001 People’s Republic of China; 2 Department of Orthopedics, Joint Surgery and Sports Medicine, First Affiliated Hospital of China Medical University, 155 Nanjing North StreetLiaoning Province, Shenyang, 110001 People’s Republic of China

**Keywords:** PCL reconstruction, Internal brace, Clinical outcome, Return to sports

## Abstract

**Purpose:**

We developed an augmentation technique for PCL reconstruction with independent internal brace reinforcement and evaluated the functional outcome after PCL reconstruction employing autologous hamstrings augmented with an internal brace system for patients with isolated or combined grade 3 posterior instability who were treated with this technique.

**Methods:**

From January 2016 to January 2018, patients with isolated or combined grade 3 PCL tears who underwent single-bundle PCL reconstruction using autologous hamstrings augmented with independent internal braces were studied. The function of the operated knee was evaluated according to the International Knee Documentation Committee (IKDC) score, Lysholm score, and Tegner activity score. The patients were asked the level of returned to their previous sport. Posterior knee laxity was examined with a KT-1000 arthrometer, and data on range of motion (ROM), re-operation, and other complications were collected.

**Results:**

A total of 33 consecutive patients who received single-bundle PCL reconstruction using autologous hamstrings augmented with independent internal braces with a minimum two years follow-up were included in this study. Two patients had undergone this procedure during the study period and were not included in this study (one had combined bone fractures, and one patient had previous meniscus surgery). Thirty-one patients were available for final analysis. The mean follow-up was 45.35 ± 10.88 months (range 29–66 months). The average IKDC subjective knee evaluation scores from 51.65 ± 12.35 to 84.52 ± 6.42, the Lysholm score from 53.90 ± 11.86 to 85.68 ± 4.99, and the Tegner score from 2.81 ± 0.79 to 6.71 ± 1.83 (*P* < 0.05 for all). The mean total posterior side-to-side difference in knee laxity, assessed using a KT-1000 arthrometer, decreased from 12.13 ± 2.66 mm pre-operatively to 1.87 ± 0.56 mm post-operatively at 70° (*P* < 0.05). Most patients (29/31) had normal or near normal knee ROM post-operatively; two patients revealed a 6–15° loss of knee flexion compared with the contralateral knee. Twenty-nine patients (93.55%) returned to a normal daily exercise level. Twenty-three patients (74.19%) returned to competitive sports with high-level sports (Tegner score of 6 or above; eleven patients (35.48%) reported to be on the same level as well as the Tegner level); six patients (19.35%) returned to recreational sports (Tegner score of 4 or 5). Two patients had Tegner scores of 2 and 3, indicating poor function level. No patient needed PCL revision surgery during the follow-up period.

**Conclusion:**

Single-bundle PCL reconstruction with internal brace augmentation for PCL injury exhibited satisfactory posterior stability and clinical outcomes in patients with isolated or combined grade 3 PCL injuries at a minimum two year follow-up.

**Supplementary Information:**

The online version contains supplementary material available at 10.1007/s00264-022-05448-4.

## Introduction

The posterior cruciate ligament (PCL) is the primary restraint to posterior tibial translation [1–3]. PCL deficiency is known to lead to pain or impaired function and the development of degenerative changes over the long term [2, 4]. PCL injury accounts for up to 20% of injuries to the ligament around the knee [1–3, 5]; however, the optimal treatment for grade 3 PCL tears remains controversial [2, 4, 6–15]. Isolated grade 1–2 PCL rupture can be treated nonoperatively due to the good self-healing capacity of the PCL [1, 2]. Patients with isolated grade 3 PCL tears who fail conservative treatment or have other coexisting knee ligament injuries usually require surgical treatment [2, 4, 6–15].

PCL reconstruction surgery remains the most common method for treating complete PCL tears [1, 4, 5, 9–16]; however, there is no consensus on which PCL reconstruction technique is optimal. Controversies remain regarding the timing of surgery, graft choice, type of reconstruction (single-bundle vs. double-bundle), and technique (transtibial vs. tibial inlay) [2, 3, 8, 12, 14–20]. PCL reconstruction often leads to inferior results when compared to anterior cruciate ligament (ACL) reconstruction [2, 15, 21, 22]. Persistent knee laxity after reconstruction is often reported, and the graft failure rate of PCL reconstruction has been reported to be fairly high; nearly 5–21% of patients need to undergo PCL revision [2, 6, 21–27].

Improved understanding of native biomechanics along with enhanced implant technology (such as internal bracing technology) could ultimately improve the biomechanical characteristics of PCL reconstruction [28]. Recently, cruciate ligament reconstruction or repair augmented with independent suture tape using high strength (i.e., an internal brace system) has been proposed [20, 28–30]. The theoretical advantage of internal bracing technology is that it improves the biomechanical characteristics of PCL reconstruction [20, 28]. However, to the best of our knowledge, the clinical outcomes of the internal brace augmentation technique for PCL reconstruction have not been reported.

We have performed PCL reconstruction with suture tape augmentation with an internal brace since 2016. The purpose of this study was to evaluate the functional outcome after PCL reconstruction employing autologous hamstrings augmented with an internal brace system for patients with isolated or combined grade 3 posterior instability.

## Methods

### Patients

**Fig. 1:**
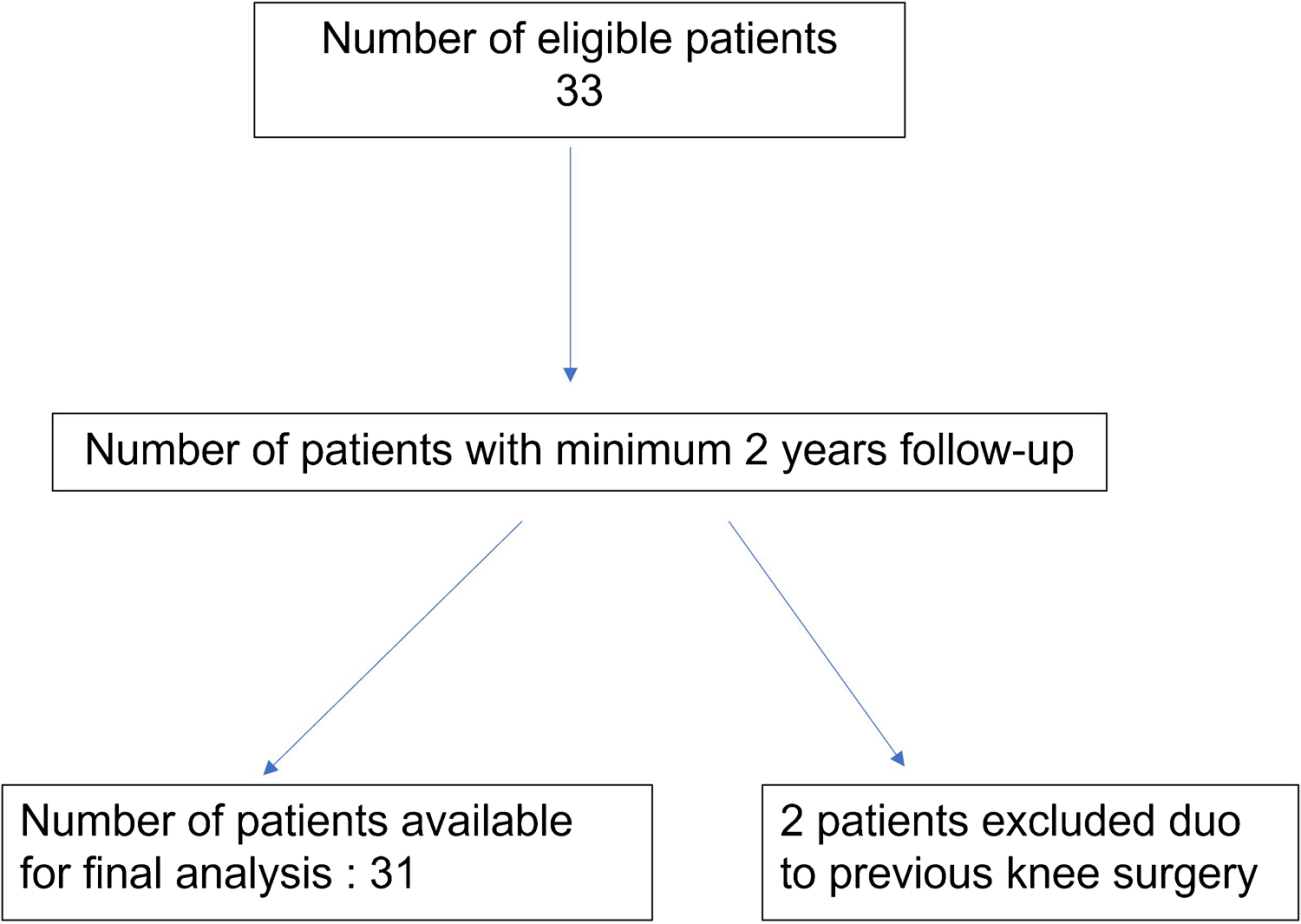
CONSORT flowchart of this trial

**Fig. 2:**
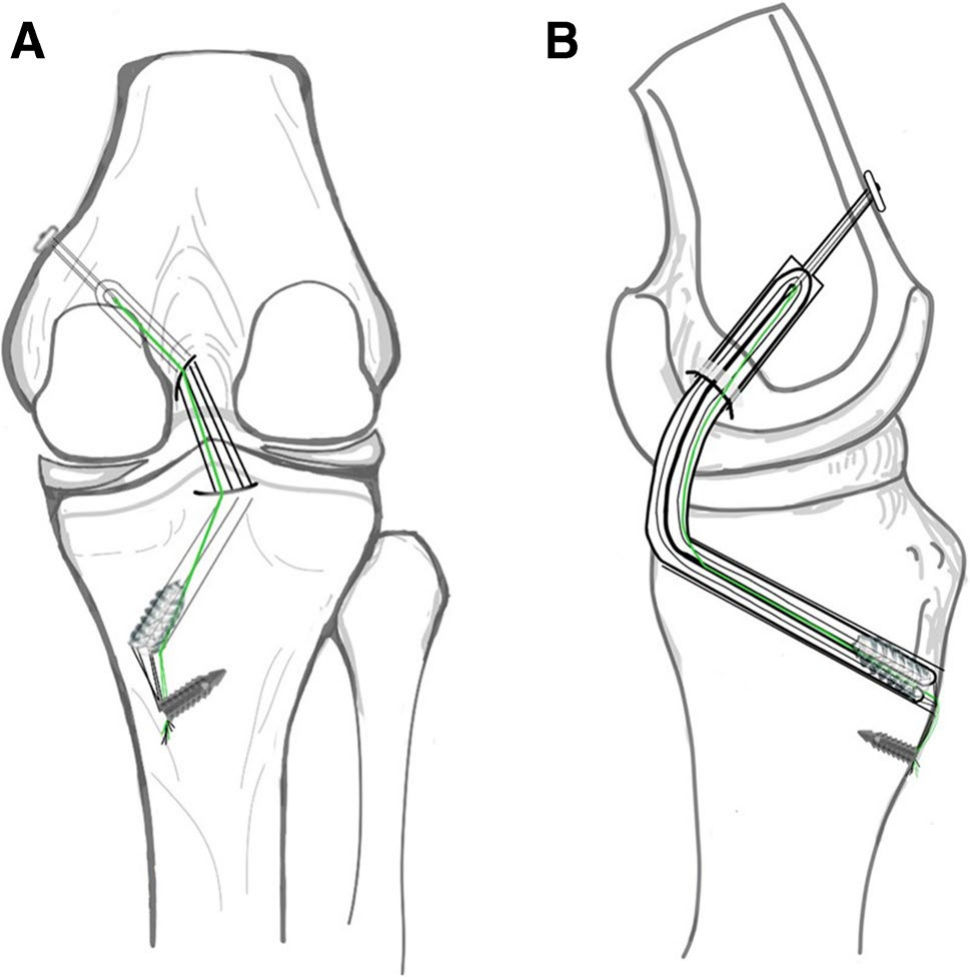
Illustration of the technique (right knee) used for PCL reconstruction augmented with an internal brace (**A**, **B**)

**Fig. 3:**
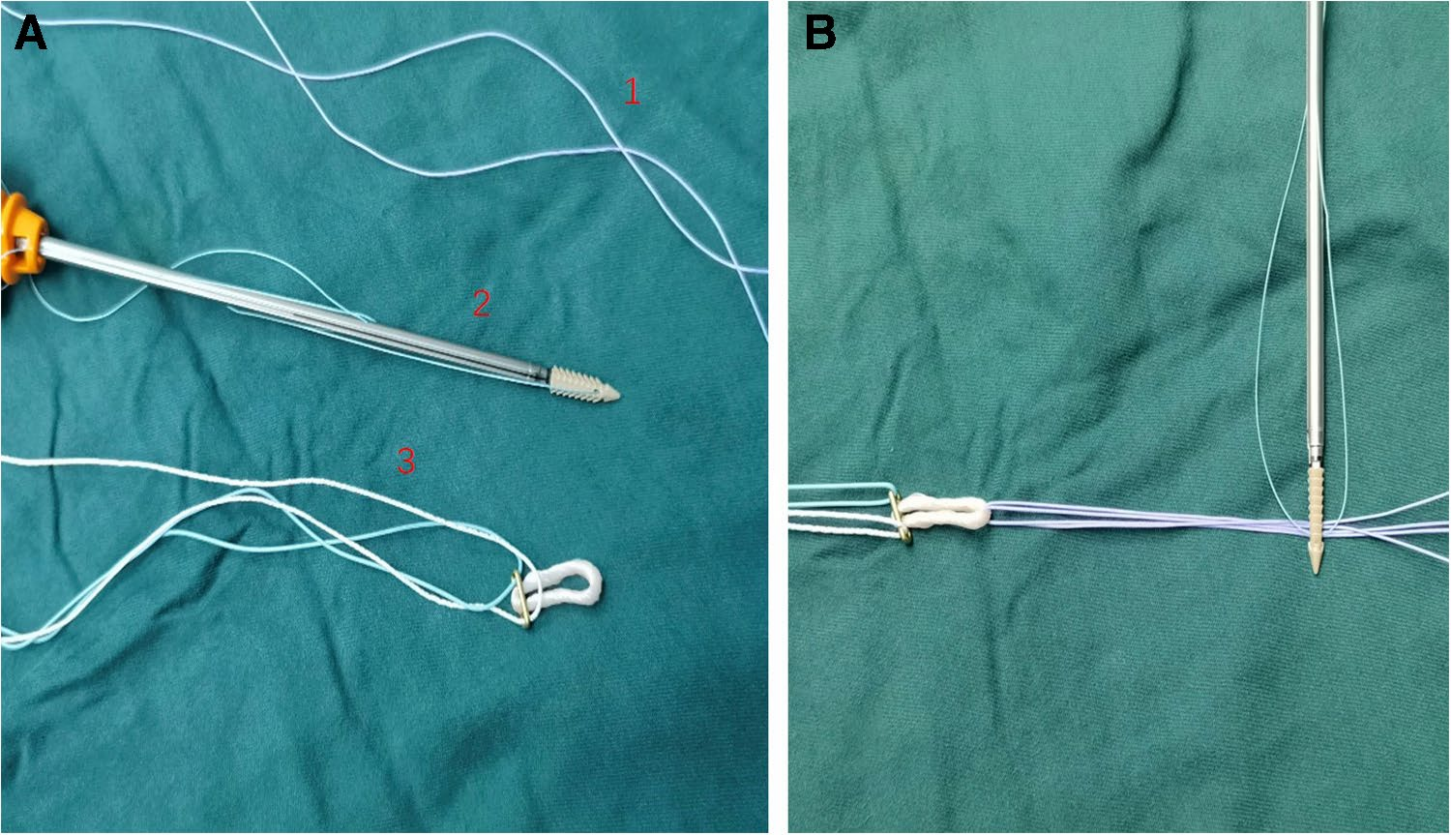
Example of an internal brace. **A** 1, No. 2–0 OrthoCord (DePuy Mitek, Raynham, MA); 2, a fixed 20-mm ENDOBUTTON (Smith & Nephew); 3, a FOOTPRINT ULTRA PK suture anchor (4.5 mm) (Smith & Nephew). **B** Internal brace ligament augmentation

We retrospectively collected data between January 2016 and January 2018 on patients with isolated grade 3 PCL injuries or combined injuries (Fig. 1). All patients who underwent PCL reconstruction with hamstring tendon autografts and the transtibial tunnel technique augmented with an internal brace system using high-strength sutures (Figs. 2 and 3) and who had a minimum follow-up of two years were included in this study.

The exclusion criteria were as follows: (1) combined bone fracture on the affected leg, (2) active infection (septic arthritis or soft-tissue infection), and (3) any previous surgery on the affected knee. The patients were invited to participate in the study. Ethics approval was approved by the local ethics committee (No. AF-SOP-07–1.1–01). Informed consent was obtained from each patient enrolled in the study. A total of 33 patients who underwent post-operative follow-up were included in this study (Table 1). Two patients had undergone this procedure during the study window period and were not included in this study.

**Table 1:** Distribution of ligament reconstruction in this case series

Ligament reconstruction in patient material	Total	Mean age at surgery (range)	Sex (M/F)
PCL isolated	14	35.79 ± 14.44	8/6
PCL + ACL	5	36.1 ± 8.44	3/2
PCL + ACL + MCL	3	33.33 ± 7.03	3/0
PCL + MCL	3	37.67 ± 2.08	2/1
PCL + ACL + PLC	2	41.5 ± 4.95	2/0
PCL + PLC	4	33.5 ± 4.65	3/1
Total	31	35.55 ± 10.48	21/10

The final IKDC and Lysholm scores were recorded in January 2020 at the latest follow-up. Fourteen of 31 patients underwent isolated posterior cruciate ligament (PCL) reconstruction. The rest underwent PCL construction combined with other ligament reconstruction (ACL reconstruction, PLC reconstruction) or repair (MCL repair). *F*, female; *M*, male; *ACL*, anterior cruciate ligament; *MCL*, medial collateral ligament; *PCL*, posterior cruciate ligament; *PLC*, posterolateral corner

### Surgical technique

Examination under anesthesia was performed to confirm any pathology, such as in the menisci, cartilage, and cruciate ligaments. If needed, meniscal or cartilage surgery was also performed (8 patients underwent meniscal surgery, and 2 patients underwent cartilage surgery).

### PCL reconstruction

In our study, all PCL reconstructions were reconstructed via transtibial technique-assisted single-bundle PCL reconstruction using an autograft independently augmented with suture tape. ACL reconstruction, PLC reconstruction, and/or medial collateral ligament (MCL) repair were also performed in the same anesthetic session. The graft choice was based on the type of instability (Table 2).

**Table 2:** Graft choice in one-stage reconstruction for isolated grade 3 PCL injury or grade 3 PCL injury combined with multiligamentous knee injury

Reconstruction	Autograft selection			
	ACL	PCL	MCL	PLC
Isolated PCL	-	Ipsilateral STG	-	-
PCL + MCL	-	Ipsilateral STG	MCL repair or conservation	-
PCL + ACL + MCL	Contralateral STG	Ipsilateral STG	MCL repair or conservation	-
PCL + PLC	-	Ipsilateral STG	-	Contralateral STG
PCL + ACL + PLC	Contralateral STG	Ipsilateral STG	-	Ipsilateral peroneal tendon

*STG*, semitendinosus tendon; *ACL*, anterior cruciate ligament; *MCL*, medial collateral ligament; *PCL*, posterior cruciate ligament; *PLC*, posterolateral corner

## Graft preparation and internal brace preparation

**Fig. 4:**
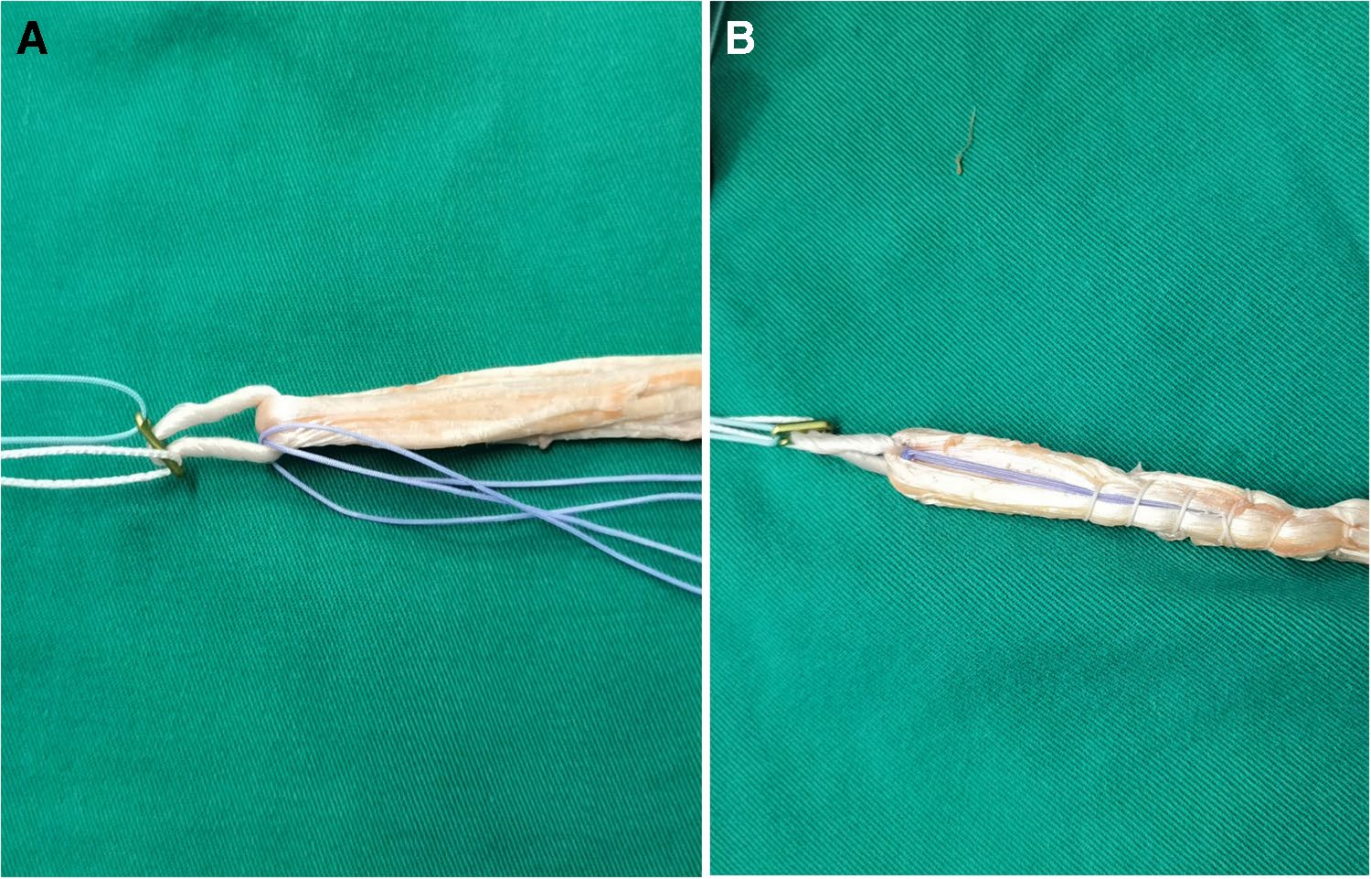
The doubled tendons and 2 doubled high-strength sutures combined in advance (**A**, **B**)

Two No. 2–0 OrthoCord (DePuy Mitek, Raynham, MA) were looped through the suspensory device (15 mm ENDOBUTTON, Smith & Nephew) and incorporated into the graft with an autograft, which was folded into a 12 cm, four-strand tendon graft (Figs. 3 and 4). The tendons were sutured using No. 2 nonabsorbable sutures (ULTRABRAID; Smith Nephew).

### Femoral tunnel preparation

The femoral guide pin was directed at the 1:30 (right knee) or 10:30 (right knee) position. The femoral tunnel was created with an outside technique. The femoral guide pin was overreamed with a 4.5-mm ENDOBUTTON drill. The femoral bone socket was enlarged to the measured graft size (8–9 mm in diameter, according to the diameter of the grafts).

### Tibial tunnel preparation

After routine arthroscopic examination, a posteromedial portal was created to identify the tibial PCL attachment. The creation of tibial tunnels began with the use of an appropriate guide system (Smith & Nephew). The guide was inserted through the anteromedial portal, and the guide tip was placed 10 to 12 mm below the joint line in the PCL facet. The drill guide was oriented approximately 60° to the articular surface of the tibia, starting just inferior and medial to the tibial tuberosity. A guide pin was drilled from the anterior to the posterior and exited through the center of the original PCL tibial footprint. The chosen site was the center of the PCL footprint, and the drill was advanced under direct vision to minimize the risk of neurovascular injury. An 8–9 mm reamer was drilled over the tibial guide pin to create the tibial tunnel.

### Graft passage and fixation

**Fig. 5:**
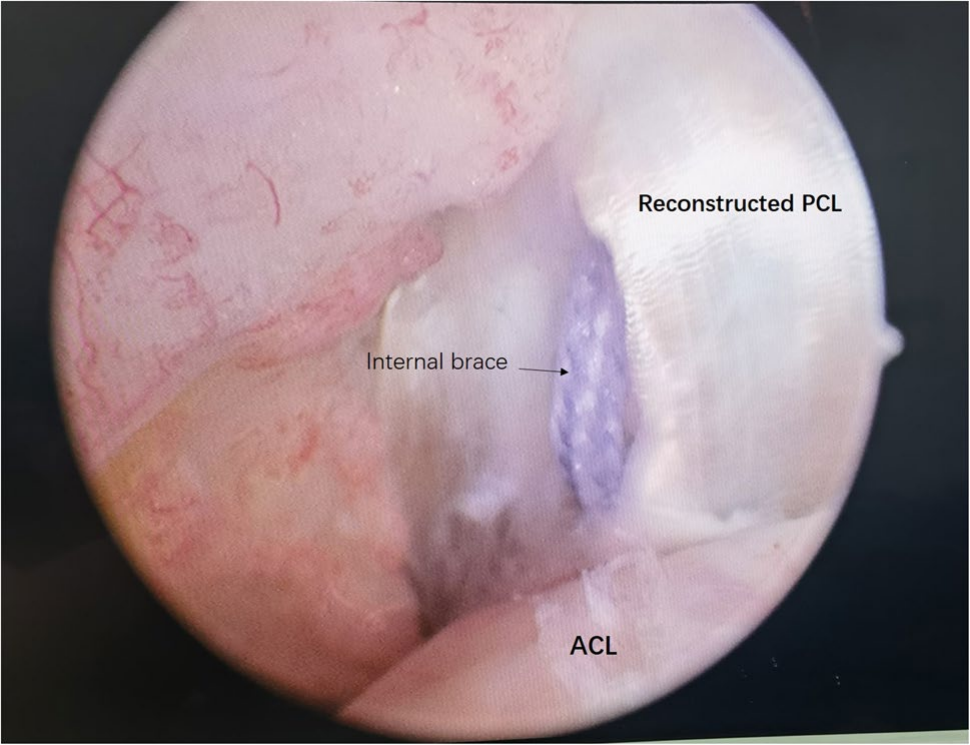
The graft was placed with high-strength sutures and exposure of the joint; the intraoperative photograph shows suture tape augmentation (internal brace)

The graft was passed intraarticularly into the tibial tunnel and femoral tunnel with the aid of a wire loop. Graft fixation was performed with an ENDOBUTTON on the femoral side first. The graft was cycled several times before final fixation on the tibial side to minimize graft elongation. The graft was fixed on the tibial side while holding the knee in 70 to 90° of flexion and applying an anterior drawer force to obtain a proper anatomic position. At our institution, tibial fixation is performed with an interference screw. FiberTape was secured to the tibia using a FOOTPRINT Ultra PK suture anchor (4.5 mm) (Smith & Nephew). This so-called internal brace augmentation was used to achieve additional pullout strength and achieve higher stiffness on the tibial side (Figs. 2 and 5). We chose to avoid using the interference screw alone for soft-tissue fixation, avoiding decreased pullout strength with cyclic loading.

#### ACL reconstruction

Reconstruction of the ACL was performed using the single-bundle technique. The centre of the femoral tunnel was placed in the 2 o’clock position for the left knee and the 10 o’clock position for the right knee; a 2-mm guide pin was inserted to the centre of the tibial footprint, approximately 2 to 3 mm anterior to the posterior margin of the anterior horn of the lateral meniscus and 7 mm anterior to the PCL. The femoral side of the reconstructed ACL was fixed with a 20-mm fixed ENDOBUTTON (Smith & Nephew). The tibial side was fixed with an interference screw (Smith & Nephew).

#### MCL repair/PLC reconstruction

In cases where MCL repair was needed, in our study, the MCL was repaired with double 4.75-mm suture anchors with No. 2 polyethylene sutures (ULTRABRAID®). The lateral collateral ligament (LCL) and posterolateral corner (PLC) were reconstructed as described by Jakobsen et al. [8].

### Post-operative rehabilitation

Post-operatively, all patients were immobilized in a functional brace for six to eight weeks. Exercises included straight leg raises, quadricep setting exercises, and calf pumps, which were encouraged beginning the day after surgery. For isolated PCL reconstruction, the operated knee was protected in a post-operative brace with 0–30° of motion permitted during the first two weeks. From two to four weeks, the operated knee was permitted 0–60° of motion. From four to six weeks, partial weight bearing was permitted with 0–90° of motion. Six weeks post-operatively, full range of motion (ROM) knee flexion was encouraged as tolerated. From six to eight weeks, partial weight bearing was permitted. Controlled sports activities (such as slow walking, biking, or swimming) could be performed from three months post-operatively, depending on the activity. Running was allowed at six to nine months. Return to contact sports was allowed nine months post-operatively. For PCL reconstruction combined with ACL reconstruction, MCL repair, or LCL repair, post-operative rehabilitation was more restrictive. In general, the motion of the knee was restricted to 0–30° for the first two weeks. From three to six weeks, the operated knee was allowed 0–60° of motion. From six to eight weeks, partial weight bearing was permitted with 0–90° of motion. After two months, the full range of knee flexion was encouraged, and standing and walking were permitted. Free activity without a brace was performed beginning at nine weeks. Controlled sports activities were allowed after six months, and return to contact sports was allowed after 12 months post-operatively.

### Evaluation

Both pre-operative and post-operative knee evaluations were performed by an experienced orthopaedic surgeon (Duan) who was blinded and not involved in the surgery and who independently examined the patients and evaluated knee function through clinic visits.

**Fig. 6:**
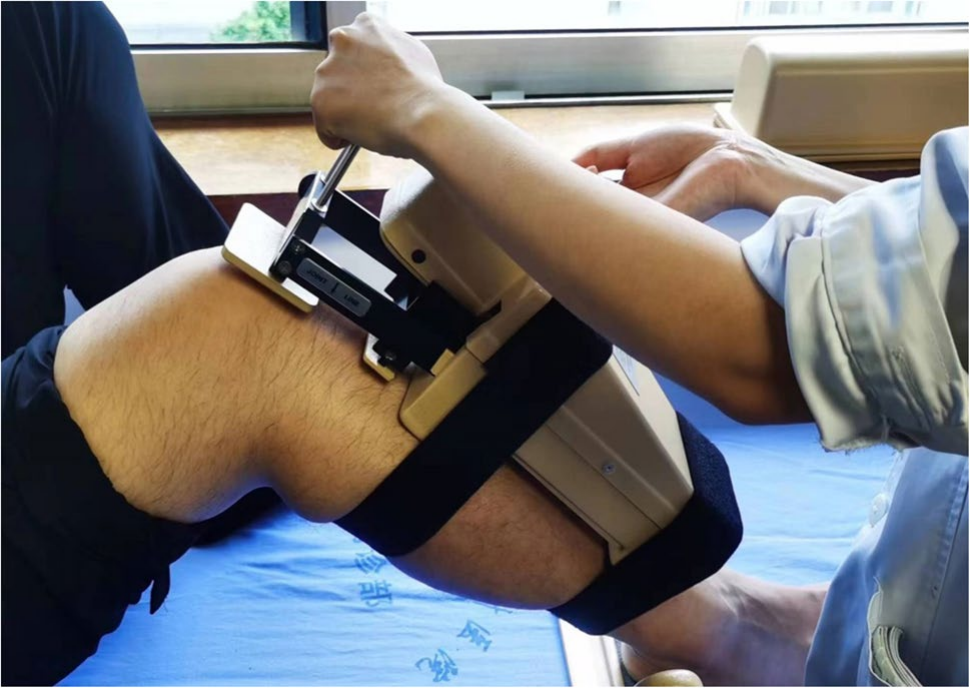
The KT-1000 arthrometer, with70° trunk-thigh flexion angle (sitting)

**Fig. 7:**
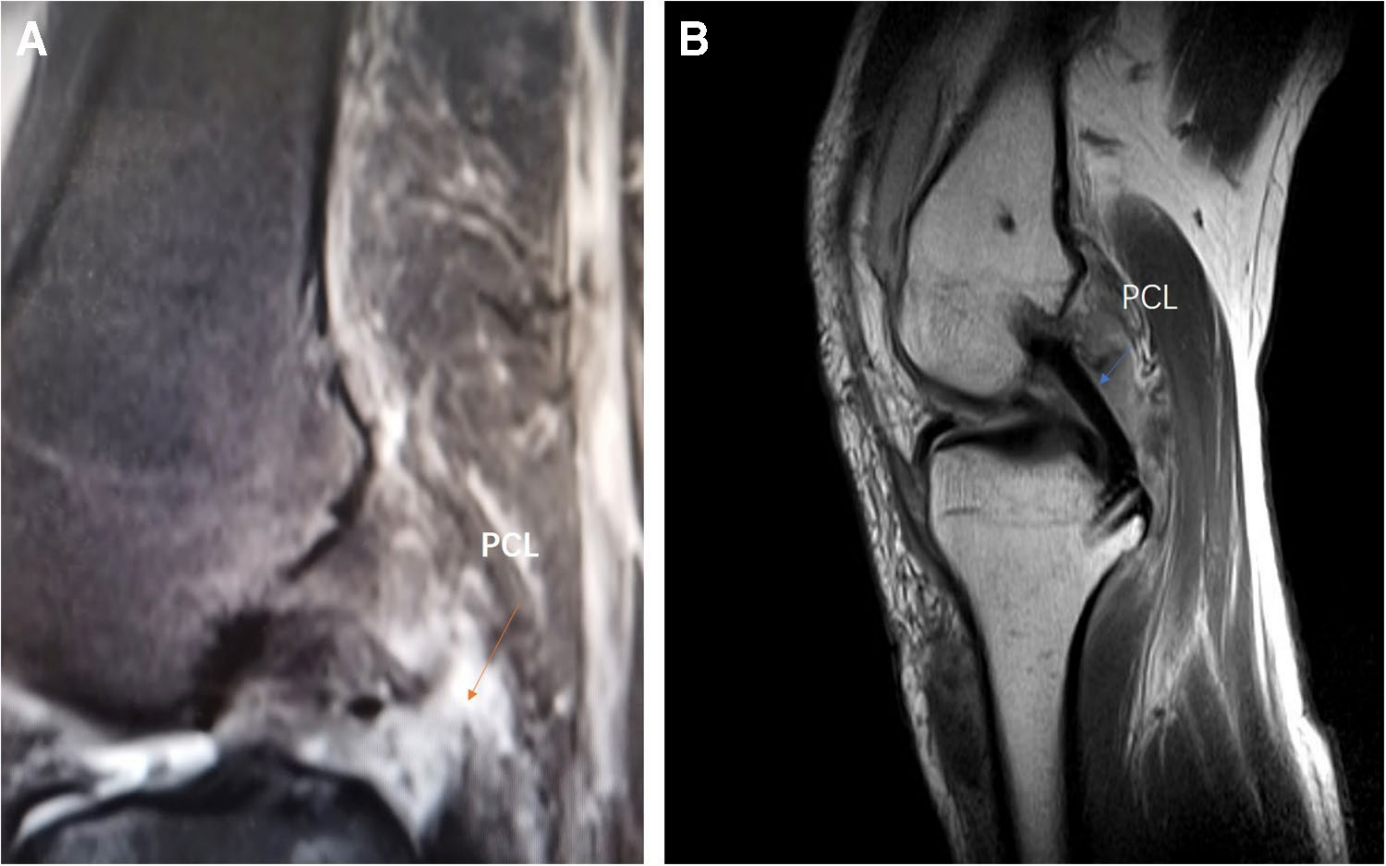
The appearance of the PCL reconstruction on magnetic resonance imaging (MRI) is shown. **A** Pre-operatively, a complete PCL injury was visible on MRI. **B** The reconstructed PCL is shown on post-operative 12-month MRI

The follow-up evaluations were performed by the same examiner (M. Y.), who was not involved in the treatment of these patients through clinic visits. The knee condition during the preoperative and last follow-up (2021) periods was evaluated based on side-to-side differences between the injured and uninjured legs. Because of the focus on posterior stability, posterior knee laxity was measured using a KT-1000 arthrometer (MEDMetric, San Diego, CA, USA) with an applied posterior force of 134 N at 70° of knee flexion (Fig. 6) (side-to-side difference; grade 1 (< 5 mm), grade 2 (5 to < 10 mm), or grade 3 (> 10 mm)). Anterior knee laxity was also measured with the KT-1000 arthrometer with an applied posterior force of 134 N and 70° of knee flexion (side-to-side difference; grade 1 (< 5 mm), grade 2 (5 to < 10 mm), or grade 3 (> 10 mm)). Valgus and varus instability was evaluated with abduction and adduction stress tests in both 0° and 30° of flexion and categorized as negative, 1 + , 2 + , and 3 + according to the IKDC criteria (grade 1 (< 5 mm), grade 2 (5 to < 10 mm), or grade 3 (> 10 mm)). The knee ROM was also assessed both pre-operatively and post-operatively. Pre-operative and post-operative magnetic resonance imaging (MRI) was performed to evaluate the patients with knee ligaments (Fig. 7). Both pre-operatively and post-operatively, functional outcome scores, including ROM, the IKDC subjective knee score[31], the Lysholm knee score[32], and the Tegner score[33], were applied to evaluate knee function. Any complications and re-operations were documented.

### Statistical analysis

Statistical analysis was performed using SPSS 18.0 (SPSS, Chicago, IL, USA). Paired *t* tests were used to compare pre-operative and post-operative IKDC scores, Lysholm scores, and posterior drawer and manual valgus test results. In all analyses, *P* < 0.05 indicated a statistically significant difference.

## Results

This study included 33 patients, 21 men and 12 women. Two patients were excluded from this study: one had combined bone fractures, and one patient had previous meniscus surgery (Fig. 1). The mean patient age was 35.55 ± 10.48 years (range, 18–53) at the time of surgery. The main cause of injury was sports-related injury in 13 patients, work-related injury in nine patients, and motor vehicle accident in nine patients. Fourteen patients underwent isolated PCL reconstruction, five underwent combined ACL reconstruction, four underwent combined PLC reconstruction, three underwent combined MCL repair, and five underwent multiple ligament reconstructions. Table 1 summarizes the general patient information. The mean follow-up time was 45.35 ± 10.88 months (range 29–66 months).

### Knee stability

**Fig. 8:**
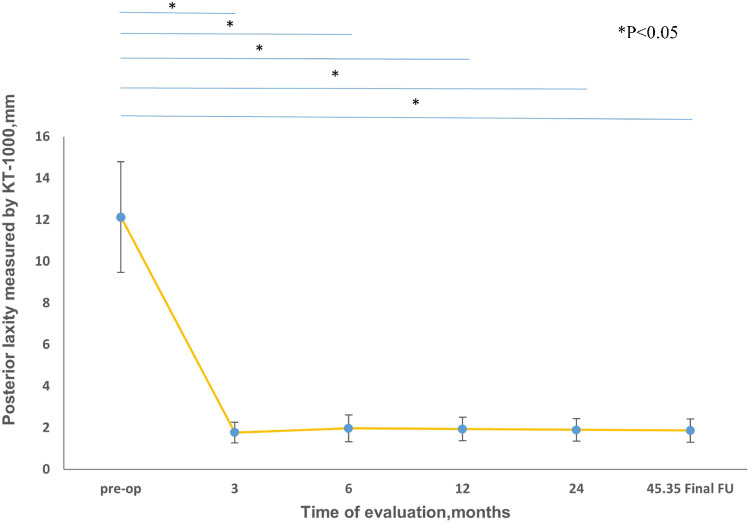
Posterior laxity measured by KT-1000 arthrometer. There is a statistically significance between the pre-operative posterior laxity and post-operative posterior laxity. FU, follow up

The posterior drawer test results at 0° and 30° were both significantly improved at the final follow-up. The posterior drawer test was also negative in 30 out of 31 patients; however, 1 + instability remained in one patient. The mean side-to-side difference in posterior laxity was also assessed using a KT-1000 arthrometer. The mean side-to-side difference improved from 12.13 ± 2.66 mm pre-operatively to 1.87 ± 0.56 mm at the last follow-up (Fig. 8) (*P* < 0.05). No patient had grade 2 or grade 3 posterior instability post-operatively, whereas 100% of patients had this problem pre-operatively. Valgus laxity at 30° was 3 + in five patients pre-operatively and became negative in four patients and 1 + in one patient post-operatively. Varus laxity at 30° was 3 + in three patients and 2 + in five patients pre-operatively and became negative in seven patients and 1 + in one patient post-operatively.

According to the MRI evaluation, the scans showed PCL injury (Fig. 7A). At follow-up, in patients who were available for post-operative MRI evaluations (*n* = 23), the reconstructed PCL could be seen as continuous low-signal bands of fiber on sagittal T_2_-weighted MRI scans (Fig. 7B).

### Subjective evaluation and return to sports

All patient-reported outcome scores (IKDC score and Lysholm score) improved pre-operatively to final follow-up (Table 3). The mean Lysholm score improved from 53.90 ± 11.86 pre-operatively to 85.68 ± 6.84 post-operatively (*P* < 0.05). The mean IKDC score improved from 51.65 ± 12.35 pre-operatively to 84.52 ± 6.42 points post-operatively (*P* < 0.05). The Tegner score from 2.81 ± 0.79 to 6.71 ± 1.83 (*P* < 0.05). Twenty-nine patients (93.55%) returned to a normal daily exercise level. Twenty-three patients (74.19%) returned to competitive sports with high-level sports (Tegner score of 6 or above; eleven patients (35.48%) reported to be on the same level as well as the Tegner level); six patients (19.35%) returned to recreational sports (Tegner score of 4 or 5). Two patients had Tegner scores of 2 and 3, indicating poor function level. Of the two patients who returned to poor function level, one underwent MCL repair and PCL reconstruction, and one underwent ACL + PCL reconstruction + MCL repair.

**Table 3:** Clinical results: comparison of outcome parameters (pre-operative and final follow-up)

	**Preoperative**	**Final follow-up**	***P* value**
IKDC score	51.65 ± 12.35	84.52 ± 6.42	< 0.05
Tegner score	2.81 ± 0.79	6.71 ± 1.83	< 0.05
Lysholm score	53.90 ± 11.86	86.68 ± 6.84	< 0.05
KT-1000 measurements for posterior stability (mm)	12.13 ± 2.66	1.87 ± 0.56	< 0.05

Data are presented as the mean and standard deviation. *IKDC*, International Knee Documentation Committee

### Range of motion and other complications

Pre-operatively, all patients had full ROM. At the follow-up, ROM deficits were seen in two of the 31 patients. One patient had a deficit of 10° of flexion at the last follow-up; however, this patient was satisfied with the post-operative results and refused manipulation of the knee to regain normal motion of the knee. Another patient in this study required manipulation under anaesthesia due to loss of 15° of knee flexion. During the follow-up period, one patient underwent additional surgery procedures. This patient returned for lateral meniscectomy of the operated knee two years post-operatively. No patient needed revision PCL surgery. There were no post-operative infections or iatrogenic neurovascular injuries in our patients.

## Discussion

The most important finding of the present study was that patients who underwent PCL reconstruction augmented with internal braces improved in terms of posterior stability and subjective knee function postoperatively. In this paper, we present our results using this technique with 31 patients. The mean side-to-side difference in knee laxity assessed using the KT-1000 arthrometer was significantly reduced to 1.87 mm at a minimum two year follow-up compared with 12.13 mm pre-operatively. No patient had grade 2 or grade 3 posterior instability post-operatively, whereas 100% of patients had this problem pre-operatively.

Complete PCL tears are difficult injuries to treat. The failure rate of PCL reconstruction has been reported to be fairly high, and nearly 5–30% of patients need to undergo PCL revision [6, 21, 23–27]. Current techniques for PCL reconstruction utilize either a transtibial approach, with potentially complicated graft passage around the killer curve in addition to the risk of vascular injury due to drilling toward the popliteal fossa, or a tibial inlay technique, with prone patient positioning, which may be cumbersome to many surgeons and increase the operative time[1, 2, 6, 24, 25]. MacGillivray et al. [16] compared the transtibial technique with the tibial inlay technique, and there were no significant differences in posterior drawer testing, KT-1000 measurements, functional test results, or Lysholm and Tegner knee scores in either group at a minimum two year follow-up. Currently, internal brace augmentation is a technique that assists ligament repair or ligament reconstruction, and it is a bridging concept that involves using braided suture tape to reinforce ligament strength and act as a stabilizer after repair or reconstruction [10, 20, 27–29]. The use of high-strength sutures is a very good functional alternative, with good results documented in the literature [10, 28, 29, 34]. van der List [30] and Hopper et al. [7] described PCL repair using internal brace augmentation but did not examine clinical outcomes. Trasolini et al. [20] presented a biomechanical study of PCL reconstruction using internal brace augmentation. This study evaluated the stiffness and resistance to elongation of an internal bracing construct in PCL reconstruction. The internal brace augmentation showed significantly less tibial translation with posterior drawer loading than the PCL reconstruction without an internal brace. No significant differences were found in overall construct stiffness between groups. Clinical studies are warranted to determine whether these ex vivo biomechanical benefits translate to improved outcomes.

It is essential to avoid graft stretching and construct lengthening to protect the healing PCL before graft incorporation [10, 20]. Grotting et al. [28] presented a biomechanical study and compared the kinematics and patellofemoral contact pressures of all inside and transtibial single-bundle PCL reconstructions to determine whether suture augmentation further improves the biomechanics of either technique. They found that in this time-zero study, suture augmentation in both techniques provided further anterior–posterior stability. Levy et al. [10] biomechanically evaluated the effect of independent suture tape (ST) reinforcement on PCL reconstruction using porcine bones and quadrupled bovine tendons. They found that adding independent suture taping to PCL reconstruction led to improvement in the studied metrics by reducing the total elongation and increasing the ultimate failure strength. The ST appears to be a “safety belt,” which becomes more dominant when the graft is exposed to higher loads, where it demonstrates more plastic deformation. They pointed out that PCL reconstruction with additional ST was able to withstand higher loads until failure than constructs without ST. Grotting et al. [28] also found that suture augmentation can provide further anterior–posterior stability. The findings in this patient series are generally in agreement with those of other studies, and this approach may more effectively reproduce the posterior stabilization of the knee [10, 20]. Our study indicates that using internal brace augmentation for PCL reconstruction is clinically useful in the treatment of PCL-deficient knees. 74.19% patients returned to competitive sports with high-level sports. 35.48% patients reported to be on the same level. The complication rate was low, and joint stability was significantly improved. No patient needed PCL revision surgery during the follow-up period. This study on PCL reconstruction augmented with independent internal brace fixation reported clinical outcomes. We believe the findings of this study add to the existing knowledge on PCL reconstruction.

There are some limitations in the present study. First, the present study is limited by the fact that it is a retrospective case series, and we have only a small number of patients (31 patients). Furthermore, there was a heterogeneous patient population and procedures, and there was a small number of patients in each of the subgroups. Second, more patients and long-term clinical outcomes after PCL reconstruction augmented with internal braces for patients with PCL laxity should be further assessed. Third, inherent limitations of the study included the possibility of information bias and the lack of a control group (other PCL reconstruction techniques). Larger randomized controlled studies are needed to review the safety of this technique compared with the standard tibial inlay and transtibial tunnel techniques. Fourth, this study included patients who had various types of multiligament injuries. Even though all patients underwent PCL reconstruction using the same technique, we recognize that it would be preferable if these different reconstructions of patients were evaluated separately. However, due to the limited number of patients in this study, we performed an overall evaluation and an individual evaluation. A comparative study with a larger number of cases and long-term follow-up is required to further evaluate the optimal treatment strategy for grade 3 PCL tears in multiligament injuries. Fifth, most patients refused X-ray examination post-operatively, and stress radiography was not included in this study. For patients who underwent LCLR or MCL repair, we evaluated valgus and varus instability with abduction and adduction stress tests, which are subjective assessments. Stress radiographic examination should have been performed to evaluate valgus and varus instability more objectively.

## Conclusions

Single-bundle PCL reconstruction with internal brace augmentation for PCL injury exhibited satisfactory posterior stability and clinical outcomes in patients with isolated or combined grade 3 PCL injuries at a minimum two year follow-up.

## Supplementary Information

Below is the link to the electronic supplementary material.

Supplementary file1 (XLSX 21 KB)
